# Duration of breastfeeding in preterm infants followed at a secondary referral service

**DOI:** 10.1016/j.rppede.2016.02.010

**Published:** 2016

**Authors:** Brunnella Alcantara Chagas de Freitas, Luciana Moreira Lima, Carla Fernanda Lisboa Valente Carlos, Silvia Eloiza Priore, Sylvia do Carmo Castro Franceschini

**Affiliations:** a Universidade Federal de Viçosa (UFV), Viçosa, MG, Brazil; b Centro Viva Vida de Referência Secundária Viçosa e Região de Saúde, Viçosa, MG, Brazil

**Keywords:** Preterm infant, Breastfeeding, Very low birth weight newborn, Extremely preterm infants

## Abstract

**Objective:**

Identify and analyze variables associated with shorter duration of breastfeeding in preterm infants.

**Methods:**

Retrospective cohort of premature infants followed up at secondary referral service in the period of 2010-2015. Inclusion: first appointment in the first month of corrected age and have undergone three or more consultations. Exclusion: diseases that impaired oral feeding. Outcome: duration of breastfeeding. A total of 103 preterm infants were evaluated, accounting for 28.8% of the preterm infants born in the municipality in that period, with a power of study of 80%. Descriptive analysis, *t*-test, chi-square test, Kaplan-Meier curves and Cox regression were used. *p*-values <0.05 were considered significant.

**Results:**

The median duration of breastfeeding among preterm infants was 5.0 months. The risk of breastfeeding discontinuation among preterm infants with gestational age <32 weeks was 2.6-fold higher than for those born at 32 weeks or more and the risk of breastfeeding interruption in preterm infants who were receiving breastfeeding supplementation in the first outpatient visit was 3-fold higher when compared to those who were exclusively breastfed in the first consultation.

**Conclusions:**

The median duration of breastfeeding in preterm infants was below the recommended one and discontinuation was associated with gestational <32 weeks and the fact that the infant was no longer receiving exclusive breastfeeding in the first outpatient visit. When these two variables were associated, their negative effect on the median duration of breastfeeding was potentiated.

## Resumo

**Objetivo:**

Identificar e analisar as variáveis associadas à menor duração do aleitamento materno em prematuros.

**Métodos:**

Coorte retrospectiva de prematuros acompanhados em centro de referência secundária, de 2010 a 2015. Inclusão: primeira consulta no primeiro mês de idade corrigida para prematuridade e ter feito três ou mais consultas. Exclusão: doenças que impossibilitassem a alimentação via oral. Desfecho: duração do aleitamento materno. Avaliaram-se 103 prematuros, 28,8% dos prematuros do município no período, com poder do estudo de 80%. Usaram-se análise descritiva, teste *t*, qui-quadrado de Pearson, curvas de Kaplan-Meier e regressão de Cox. Considerou-se significativo o *p*-valor<0,05.

**Resultados:**

A duração mediana do aleitamento materno entre os prematuros foi de cinco meses. O risco de interrupção do aleitamento materno entre prematuros de idade gestacional inferior a 32 semanas foi 2,6 vezes maior em relação aos que nasceram com 32 semanas ou mais e o risco de interrupção do aleitamento materno em prematuros que estavam em aleitamento materno complementado na primeira consulta ambulatorial foi três vezes maior em relação aos que estavam em aleitamento materno exclusivo na primeira consulta.

**Conclusões:**

A duração mediana do aleitamento materno em prematuros encontrou-se aquém do preconizado e sua interrupção se associou à idade gestacional inferior a 32 semanas e ao fato de não estar mais em aleitamento materno exclusivo na primeira consulta ambulatorial. Quando essas duas variáveis se associaram, potencializou-se sua interferência negativa na duração mediana do aleitamento materno.

## Introduction

The increase in preterm infants' survival rate has been observed in developed and developing countries. However, these infants are vulnerable to conditions leading to increased morbidity and mortality and require specialized follow-up.^1^

Decreased infant mortality and better neurodevelopment are proven benefits associated with breastfeeding.^2^^,^ 3 Breast milk is recommended as ideal for feeding preterm infants and the use of formula is only indicated when breastfeeding is impossible.^1^^,^ 4 ^,^ 5 However, preterm mothers have lower success rates in breastfeeding, which reinforces the need to adopt practices aimed to promote breastfeeding at the different levels of health care.^1^^,^ 5 ^-^ 7 In this context, the work developed at Centro Integrado Viva Vida de Referência Secundária Viçosa, established in 2010 in the state of Minas Gerais, is highlighted; the service consists of an interdisciplinary team and has become a reference for the health care of preterm infants in the city.

Based on these premises, this study aimed to identify and analyze variables associated with shorter duration of breastfeeding in preterm infants followed up in a newly implanted secondary referral service in the municipality.

## Method

This is a retrospective cohort study of data from medical records of all preterm infants followed at Centro Integrado Viva Vida de Referência Secundária Viçosa (Centro Viva Vida), in Minas Gerais, enrolled from September 2010 to June 2015. The medical records of Centro Viva Vida are semi-structured, a fact that allowed obtaining reliable data for this study.

Hospital São Sebastião, the hospital where all births take place in the municipality of Viçosa, is a reference for high-risk pregnancy and has had a milk bank since 2009 and a neonatal intensive care unit since 2004. Centro Viva Vida is dedicated to maternal and children's health and is the only preterm infants' referral service in the municipality, established in September 2010. In the service, where the health care of preterm infants is being implemented and intensified, the care is performed by the interdisciplinary team - consisting of professionals from the pediatrics, nursing, nutrition, psychology, physiotherapy and social work areas - and is linked with Universidade Federal de Viçosa. At the time of the discharge from the Hospital São Sebastião, all preterm infants are sent to Centro Viva Vida for outpatient follow-up.

**Figure 1:**
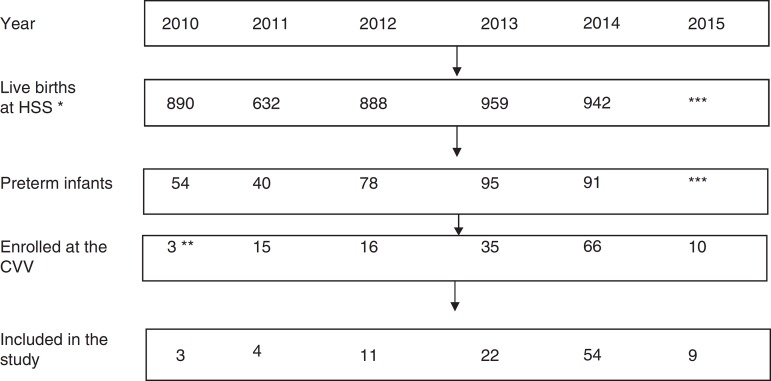
Flow chart of the of the study population selection process. Viçosa-MG, 2010-2015.*Note:* the exclusion (difference between preterm infants enrolled in CVV and included in the study) was due to the first consultation at the CVV occurring after 44 corrected weeks and/or having had fewer than three consultations; no preterm had any disease that prevented oral feeding. * All births in the municipality occur in HSS; ** CVV was establishedin September 2010; *** Data are not yet available (the study was extended to July 2015). Sources: Hospital São Sebastião (HSS) and Centro Viva Vida (CVV).

During the study period, the annual number of live births in Hospital São Sebastião, municipality of Viçosa, ranged from 632 to 959, with preterm birth rates between 6.1 and 9.9%. The details of the number of births and the total number with less than 37 weeks per each year of the study period are shown in Fig. 1.

Inclusion criteria: being born with less than 37 weeks of gestational age, with 44 weeks or less of corrected gestational age at the first consultation at Centro Viva and have undergone three or more visits during the outpatient follow-up period. Diseases that impaired oral feeding were considered exclusion criteria.

The dependent variable for the study was duration of breastfeeding measured in months, according to the corrected age.^8^

Gestational age was defined as the best estimate obtained from early pregnancy ultrasound, date of last menstrual period, obstetric data and clinical examination according to the New Ballard score.^9^^,^ 10 Chronological age was defined as postnatal age and the corrected age as the gestational age at birth plus the postnatal age.^11^

The assessed sociodemographic variables were: maternal and paternal age, maternal and paternal schooling (up to elementary school and further), maternal occupation (work outside or not), maternal marital status (single/divorced, common-law marriage/married) and family income in minimum wages (MW; <2 MW and MW≥2).^12^

The variables assessed in the prenatal and perinatal period were: type of delivery, gender, gestational age (weeks), birth weight (grams), stratification by birth weight and gestational age, adequacy of birth weight for gestational age, admission to the neonatal intensive care unit and its duration. The birth weight was categorized as <1.500g and ≥1.500g, and gestational age as <32 weeks and ≥32 weeks.^13^ The small for gestational age criterion was based on Fenton and Kim^14^ curves and was characterized as values below the 10th percentile of weight for gestational age.

The first consultation was defined as the first record in the outpatient medical file and the last consultation as the last data recorded in the medical file, of which limit was the end of the study period. The occurrence of hospitalization and the type of feeding were documented at the first consultation - categorized as exclusive breastfeeding, supplemented breastfeeding and artificial feeding^8^ - but for the bivariate and multivariate analysis, only the type of breastfeeding in the first consultation was considered (exclusive or supplemented). Anthropometric measurements in the first and last consultations were also recorded and the corrected age was also considered: weight, stature (length/height) and body mass index for age. The anthropometric measurements were collected by pediatricians or nutritionists during consultations using digital scales and anthropometer, and recorded in the medical records.

The minimum sample size was defined using the coefficient of variation obtained in the present study for the variable duration of breastfeeding (70%); considering 20% of variation around the mean, we obtained a minimum sample size of 50 individuals so that significant differences could be observed with a significance level of 5% and a power of study of 80%.^15^

For the descriptive analysis, means, standard deviations, medians, minimum and maximum values, frequencies and absolute numbers were determined. The Kolmogorov-Smirnov test was used for the continuous variables and the ones with parametric distribution were expressed as mean and standard deviation, while those with non-parametric distribution were expressed as medians and minimum and maximum values. The frequencies referred to the total number of valid responses and the missing data were not considered. A comparative analysis of the preterm infants included and those not included in the study was performed by *t* test and Pearson's chi-square test.

The nonparametric Kaplan-Meier method was used to estimate the distribution of preterm infants according to the duration of outpatient follow-up and to estimate the median duration of maternal breastfeeding. Both variables remained as continuous and were measured in months and the corrected age was considered. The Kaplan-Meier method of survival curves allowed the inclusion of censored data for the dependent variable “breastfeeding duration” (preterm infants who stopped being followed and were still being breastfed). The analysis of association between independent variables and the dependent variable was carried out in two stages. The independent variables were dichotomized according to a probable association with breastfeeding duration, with code 1 being used for the category associated with possible shorter breastfeeding duration and code 2 being used for possible longer breastfeeding duration. The bivariate analysis was used in the first stage, where we investigated the association between independent variables and the duration of breastfeeding using LogRank and Breslow tests, which compare the survival curves in each category of the independent variable. The null hypothesis in both tests is the equality of survival functions. The Cox regression model was used to analyze breastfeeding duration, adjusted for covariates. All variables that had a *p*-value <0.20 in either of the two tests of the bivariate analysis were included in the multivariate analysis. The final model included significant variables at the 0.05 level. The Statistical Package for the Social Sciences (SPSS) version 20.0 was used for the statistical analysis.

The study was approved by the Institutional Review Board of Universidade Federal de Viçosa (CAAE 19676613.5.0000.5153), as part of the project “Growth, development and morbidities of preterm or low-birth weight infants and preschool children”, which comprises a doctoral thesis developed at the Postgraduate Program in Science of Nutrition, Department of Nutrition and Health of UFV.

## Results

The medical records of 145 preterm infants were assessed and, after applying the inclusion and exclusion criteria, the study population included 103 subjects (Fig. 1), 28.8% of the preterm infants of the municipality in the period. As Centro Viva Vida (CVV) was established in September 2010, one observes a tendency to an increase in the number of preterm infants referred for outpatient follow-up after 2013. The exclusion of preterm infants was due to the first consultation at CVV after 44 corrected weeks and/or having had fewer than three consultations, as no preterm infant had any disease that prevented oral feeding.

Sociodemographic and perinatal characteristics of the preterm infants are described in Table 1, as well as the comparative analysis of those included and not included in the study. There were no significant differences between the characteristics of the studied population and the excluded preterm infants.

**Table 1:** Comparative analysis of sociodemographic and perinatal characteristics of preterm infants included and not included in the study. Viçosa-MG, 2010-2015.

Variables	Study population (*n*=103)			Excluded (*n*=42)		*p*-value
	*n* (%)	Mean±SD		*n* (%)	Mean±SD	
GA (weeks)	103 (100)	33.0±3.0		42 (100)	33.3±3.0	0.579
BW (g)	103 (100)	1913.7±635.5		42 (100)	1918.26±662.7	0.970
Maternal age	103 (100)	25.7±6.3		38 (90.4)	27.5±7.9	0.237
Paternal age	90 (87.3)	28.4±7.7		35 (83.3)	31.1±9.8	0.129
Born SGA	16 (15.5)			9 (21.4)		0.359
Cesarean delivery	67 (66.3)			27 (65.9)		0.956
Male gender	56 (54.4)			20 (47.6)		0.460
Admission to NICU	70 (68.0)			27 (65.9)		0.808
Family income <2 MW	49 (52.7)			16 (48.5)		0.678
Maternal education (≤elementary)	36 (36.0)			10 (28.6)		0.425
Paternal education (≤elementary)	47 (53.4)			15 (46.9)		0.526
Maternal occupation (works out of the home)	78 (75.7)			34 (81.0)		0.496
Maternal marital status (single/divorced)	21 (27.6)			8 (29.6)		0.843

SD, standard deviation; GA, gestational age; BW, birth weight; SGA, small for gestational age; NICU, neonatal intensive care unit; MW, minimum wage.

The population of preterm infants was distributed as follows: 31.1% had birthweight <1.500g (*n*=32) and 68.9% ≥1.500g (*n*=71); 31.1% were born with gestational age <32 weeks (*n*=32) and 68.9%≥32 weeks (*n*=71). Of the 70 preterm infants that were admitted to the neonatal intensive care unit in the neonatal period, the median hospitalization time was 26.8 days (1-135 days).

**Figure 2:**
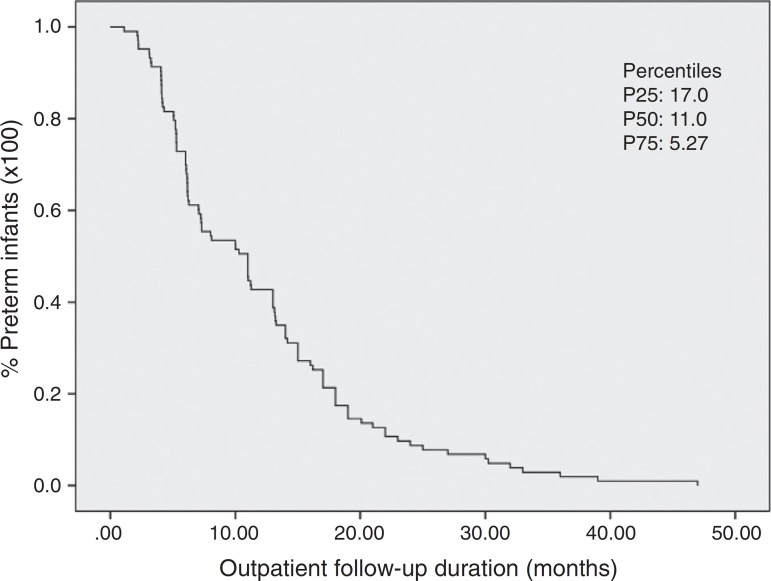
Kaplan-Meier curve with the estimate of the total time of outpatient follow-up of preterm infants (*n*=103). Viçosa-MG, 2010-2015. It can be observed that 50% of preterm infants were followed until 11 months of corrected age. The 25th and 75th percentiles are also demonstrated.

**Figure 3:**
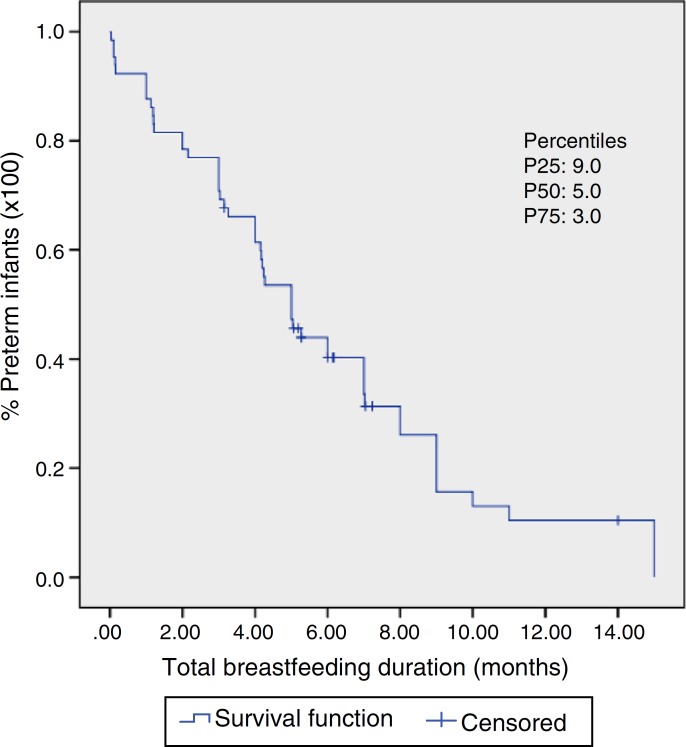
Kaplan-Meier curve with estimates of breastfeeding duration in preterm infants (*n*=71). Viçosa-MG, 2010-2015. A median of five months can be observed. The 25th and 75th percentiles are also demonstrated.

It was observed that 50% of the preterm infants were followed until 11 months of corrected age (Fig. 2). At the time of first consultation, 35.9% of the preterm infants were exclusively breastfed, 39.8% received supplemented breastfeeding and 24.3% artificial feeding (Table 2), with median breastfeeding duration of five months (Fig. 3). Among preterm infants with less than 32 weeks, 10.8% received exclusive breastfeeding (*n*=4), 26.8% supplemented breastfeeding (*n*=11) and 68% artificial feeding (*n*=17).

**Table 2:** Characteristics of preterm infants at the outpatient follow-up (*n*=103). Viçosa-MG, 2010-2015.

Variables	*n* (%)	Mean (±SD)	Median (min-max)
*First consultation*	103 (100)		
CAP (weeks, days)		40.0 (2.2)	-
Weight (g)		3124.8 (764.5)	-
Length (cm)		49.1 (3.0)	-

*Last consultation*	103 (100)		
CA (months, days)		12.1 (8.8)	-
Weight (g)		8903.0 (2411.0)	-
Height (cm)		73.6 (9.9)	-
BMI		16.2 (1.6)	-
			
*Number of consultations*	103 (100)	-	6.0 (3.0-16.0)
			
*Feeding at the first consultation*			
EBF	37 (35.9)	-	
SBF	41 (39.8)		
AF	25 (24.3)		
			
*Hospital admission*		-	-
Yes	12 (12.0)		
No	88 (88.0)		

The percentage refers to the total number of valid responses. SD, standard deviation; Min, minimum value; Max, maximum; BMI, body mass index; EBF, exclusive breastfeeding; SBF, supplemented breastfeeding; AF, artificial feeding; CA, corrected age. Causes of hospital admissions: respiratory problems (*n*=9), diarrhea (*n*=2) and inguinal hernia repair (*n*=1).

At the bivariate analysis for the breastfeeding duration outcome, through the results of the LogRank and Breslow tests, the variables family income, maternal occupation, type of delivery, birth weight, gestational age, admission at the neonatal intensive care unit and type of breastfeeding at the first consultation had a *p*<0.20 and were included for analysis in the Cox regression model (Table 3).

**Table 3:** Association between independent variables and breastfeeding duration, according to the *p*-value found in the bivariate analysis by LogRank and Breslow tests. Preterm infants, Viçosa-MG, 2010-2015.

Variables	Categories	LogRank	Breslow
Maternal education	1 - ≤Elementary	0.714	0.938
	2 - >Elementary		
Paternal education	1 - ≤Elementary	0.353	0.290
	2 - >Elementary		
Family income	1 - <2 MW	0.102	0.294
	2 - ≥2 MW		
Maternal occupation	1 - Works outside the home	0.018	0.025
	2 - Does not work outside the home		
Marital status	1 - Single/divorced	0.939	0.955
	2 - Married/common-law marriage		
Type of delivery	1 - Cesarean	0.290	0.126
	2 - Vaginal		
Birth weight	1 - <1500g	0.162	0.355
	2 - ≥1500g		
Gestational age	1 - <32 weeks	0.008	0.032
	2 - ≥32 weeks		
Born SGA	1 - Yes	0.640	0.651
	2 - No		
Admission to NICU in the neonatal period	1 - Yes	0.156	0.157
	2 - No		
Type of BF at the first consultation	1 - SBF	0.000	0.000
	2 - EBF		
Hospital admission	1 - Yes	0.599	0.434
	2 - No		

MW, minimum wage; BF, breastfeeding; SBF, supplemented breastfeeding; EBF, exclusive breastfeeding; SGA, small for gestational age; NICU, neonatal intensive care unit.

The final model for the breastfeeding duration outcome had two explanatory variables, gestational age and type of breastfeeding in the first consultation (Table 4). The adequacy of the model was verified by the development of charts involving the logarithm of the risk function versus time, and the assumption of proportional hazards was proven.

**Table 4:** Variables associated with breastfeeding duration, according to the results obtained in the multivariate analysis by Cox regression model. Preterm infants, Viçosa-MG 2010-2015.

Covariate	Coefficient	Standard error	*p*-value	Hazard ratios (95%CI)
GA	0.948	0.369	0.010	2.581 (1.253-5.317)
Type of BF at the first consultation	1.100	0.331	0.001	3.005 (1.571-5.749)

GA, gestational age; BF, breastfeeding; CI, confidence interval.

According to the LogRank and Breslow tests, the median breastfeeding duration according to the type of breastfeeding in the first consultation (exclusive or supplemented) and adjusted for gestational age (<32 weeks or ≥32 weeks) was as follows: exclusive breastfeeding in the first consultation and gestational age <32 weeks, 4.2 months; supplemented breastfeeding in the first consultation and gestational age <32 weeks, 1.2 months; exclusive breastfeeding in the first consultation and gestational age≥32 weeks, 9 months; supplemented breastfeeding in the first consultation and gestational age≥32 weeks, 4 months. The respective p-values were *p*<0.001 and *p*=0.001.

According to the Cox regression model, the following associations were found: the risk of interruption of breastfeeding among preterm gestational age <32 weeks was 2.6-fold higher when compared to those born at 32 weeks or more and the risk of breastfeeding interruption among preterm infants that were receiving breastfeeding supplementation in the first outpatient consultation was 3-fold higher when compared to those who were exclusively breastfed in the first consultation.

## Discussion

In this study, the median duration of breastfeeding among the assessed preterm infants was five months, which is below the recommended duration.^5^ Factors related to shorter breastfeeding duration were gestational age <32 weeks and supplemented breastfeeding at the first outpatient consultation. When these two variables were associated, their negative effect on the median breastfeeding duration was potentiated, which was only 1.2 months, compared with the association of exclusive breastfeeding at the first consultation and gestational age≥32 weeks, leading to a median of breastfeeding duration of nine months. Corroborating this association, the hazard ratios for breastfeeding interruption were 2.6-fold higher for preterm infants with gestational age <32 weeks and 3-fold higher for those receiving supplemented breastfeeding at the first consultation. Moreover, as negative aspects, it should be highlighted that 39.8% of preterm infants in the first outpatient consultation were receiving breastfeeding supplementation and 24.3% no longer received breast milk. The study results indicate the need to adopt strategies to establish and increase exclusive breastfeeding duration, especially of preterm infants with <32 weeks of gestational age.

The use of formula is only indicated when feeding the infant breast milk is impossible^1^^,^ 4 and, for a successful breastfeeding after discharge, it is important that the preterm infant receive exclusive breastfeeding at the time of discharge.^16^ There is evidence that mothers of preterm infants have lower success rates in breastfeeding, and thus the adoption of practices that aim to establish and maintain the supply of breast milk as first choice for feeding preterm infants, considered a vulnerable population, must be continually supported and reviewed.^1^^,^ 5 ^-^ 7 ^,^ 17

The referral hospital for the study population has a human milk bank and the family receives information and incentive to maintain lactation with frequent milking, supply of raw or pasteurized breast milk to preterm infants, skin-to-skin contact and nonnutritive sucking, handling of the transition to nutritive and breast sucking (around 34 weeks) and preparation of the preterm infant and the family to hospital discharge. These practices are recommended in literature.^1^^,^ 16 ^,^ 18 Nevertheless, lower rates of exclusive breastfeeding were observed among the studied preterm infants in the start of the outpatient follow up, when compared with supplemented breastfeeding and artificial feeding rates. There is an association between the preterm receiving supplemented breastfeeding from the first week of life and a shorter duration of breastfeeding.^19^

All preterm infants born with <32 weeks of gestational age were admitted to the neonatal intensive care unit. It is known that the health status of the preterm infant during the perinatal period is a determining factor of breastfeeding duration,^20^ but there are other factors involved, such as the insufficient stimulation of the breast and the underlying causes of preterm birth (hypertension, diabetes and maternal obesity), all of them negatively influencing breast milk production.^21^ On the other hand, studies show that maternal motivation and the support received to breastfeed are able to increase breastfeeding duration.^3^^,^ 22

The presence of human milk banks in hospitals where preterm infants are admitted is an important tool to promote breastfeeding.^23^ As effective strategies in promoting breastfeeding one can mention: providing information to the family; helping the mother to establish and maintain the milk supply; ensuring proper techniques for handling breast milk (storage and handling); development of procedures and approaches to feed preterm infants with breast milk; promoting skin-to-skin contact and nonnutritive sucking opportunities at the breast; management of the transition to breastfeeding; preparation of the preterm infant and the family to hospital discharge and appropriate follow-up.^18^ However, the establishment of the ideal time to start the transition to breastfeeding in preterm infants is still a challenge for health professionals. Frequently, unjustified delays and suction restrictions may occur due to opinions not based on evidence.^24^

A study by Wilson et al.^21^ shows that most of the mother's breast milk supply in the first week of life of preterm infants <32 weeks is associated with a 55% rate of exclusive breastfeeding rates at 36 corrected weeks for prematurity. This confirms that it is possible to achieve high rates of exclusive breastfeeding in preterm infants <32 weeks.

Although this study showed no association between hospital readmission and breastfeeding duration, preterm infants are more susceptible to readmissions and their health status affects breastfeeding duration.^20^^,^ 25

Decreased infant morbidity, mortality and better neurodevelopment are proven benefits associated with breastfeeding.^2^^,^ 3 ^,^ 26 Thus, offering follow-up and support to preterm infants and their families, especially those born at less than 32 weeks, focusing on the promotion, protection and support of breastfeeding, in order to increase exclusive breastfeeding rates, are an important issue and should be carried out in all health care levels, beginning during the hospitalization period and extending throughout the outpatient follow-up period.^20^^,^ 27 ^-^ 30 As study limitation, we mention the retrospective cohort design, subject to information biases (a fact minimized by the semi-structured model of the service medical records, which allowed a better quality of information) and changes in the sample size over time (minimized by the appropriate methodological design).

It can be concluded that, in the study, the median breastfeeding duration among preterm infants was below the recommended period and its discontinuation was associated with less than 32 weeks of gestational age and with the fact that the preterm was no longer receiving exclusive breastfeeding at the first outpatient consultation. When these two variables were associated, their negative effect on the median breastfeeding duration was potentiated. These results emphasize the need of adopting strategies to establish and increase the duration of exclusive breastfeeding, especially among preterm infants less than 32 weeks of gestational age.
